# The impact of active surveillance cultures in reducing methicillin-resistant *Staphylococcus aureus* infections in a surgical intensive care unit in Singapore

**DOI:** 10.1186/1753-6561-5-S6-P233

**Published:** 2011-06-29

**Authors:** HM Oh, T-Y Tan, GH Chua, J Li, QS Meng

**Affiliations:** 1 Medicine, Changi General Hospital, Singapore, Singapore; 2 Laboratory Medicine, Changi General Hospital, Singapore, Singapore; 3 Infection Control Unit, Changi General Hospital, Singapore, Singapore

## Introduction / objectives

Infection and colonization with methicillin-resistant Staphylococcus aureus (MRSA) is associated with significant morbidity and mortality. To study the impact of active surveillance cultures (ASC), environmental cleaning and decolonization regimen in reducing MRSA infections in Surgical Intensive Care Unit (SICU).

## Methods

The study was conducted in SICU. ASCs were performed from 20 Sep 10 to 28 Feb 11 on all patients admitted/transferred in/transferred out of SICU. ASC specimens consisted of swabs from anterior nares and axilla/groin. The swabs were inoculated onto chromogenic agar selective for MRSA (MRSASelect, Bio-Rad). MRSA positive patients were placed on contact precautions/isolation. Automatic hand sanitizers were installed in SICU to increase hand hygiene compliance. The decolonization regimen consisted of mupirocin ointment tds and daily Prontoderm (0.1% polyhexanide) for 5 days. Sureclean, an ionic silver disinfectant lasting 24 hours was used for environmental disinfection.

## Results

453 patients were screened on entry/transfer in. 45 patients (9.9%) were detected to be MRSA colonized on entry. 214 patients were screened on transfer out/death. 9 patients (4.2%) acquired MRSA on exit. There were 10 skin, 29 nasal and 15 skin/nasal carriers. There was an increase in overall hand hygiene compliance from 68.4% in Sep 10 to 90.9% in Feb 11. The incidence of MRSA Infection was reduced from 1.7/1000 patient days (Mar - Aug 10) to 0.9/1000 patient days (Sep 10 - Feb 11).

## Conclusion

We demonstrated a significant reduction of MRSA Infections in SICU with implementation of ASC.

## Disclosure of interest

None declared.

